# A 70-year-old man presenting with weakness in the left hand and tingling pain in the ulnar aspect of the left hand

**DOI:** 10.12701/jyms.2026.43.11

**Published:** 2026-01-09

**Authors:** Byungkwan Park, Ji Hwan Kim, Gyu-Sik Choi, Min Cheol Chang

**Affiliations:** 1 Yeungnam University College of Medicine, Daegu, Korea; 2 Department of Physical Medicine and Rehabilitation, Yeungnam University College of Medicine, Korea; 3 Cheokbareun Rehabilitation Clinic, Pohang, Korea

## Case presentation

A 70-year-old man was admitted to the Department of Rehabilitation Medicine of a university hospital for postoperative rehabilitation following the surgical treatment of spondylodiscitis. One month before admission, the patient was diagnosed with spondylodiscitis at the L3–L4 level, accompanied by spinal instability, for which debridement and posterior fixation from L1 to L5 were performed. The spinal surgery was performed with the patient in the prone position. He had undergone kidney transplantation for end-stage chronic kidney disease 23 years previously with ongoing oral immunosuppressive therapy consisting of tacrolimus (1 mg twice daily), mycophenolate mofetil (500 mg twice daily), and prednisolone (5 mg once daily). The patient had no additional significant medical history.

**Fig. 1.**
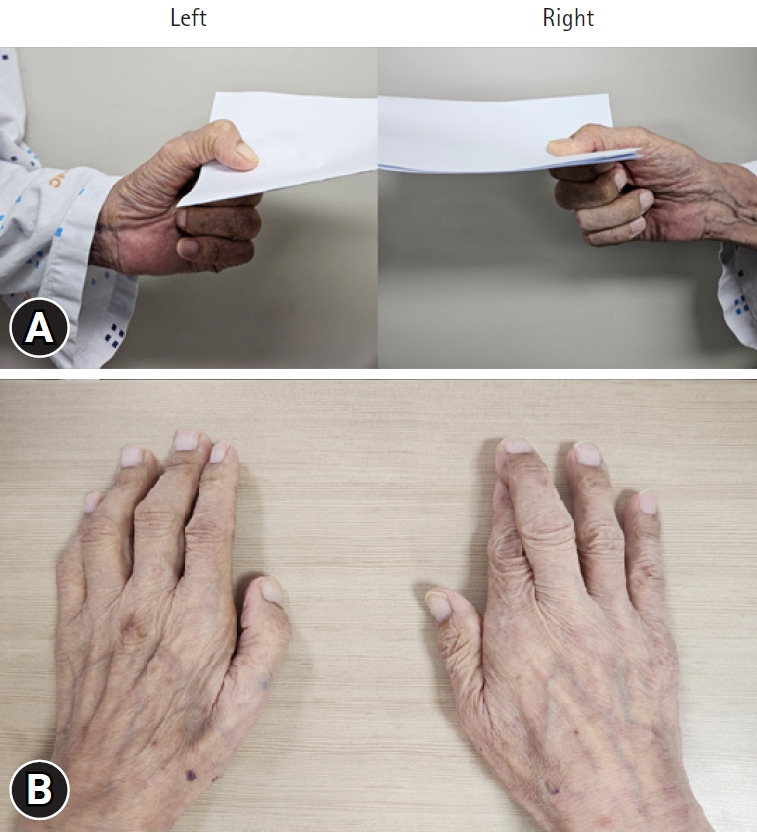
(A) Froment’s sign. The patient is instructed to hold a piece of paper between the thumb and index finger while the examiner attempts to pull it away. In ulnar nerve palsy, weakness of the adductor pollicis prevents a normal pinch, and the patient compensates by flexing the thumb interphalangeal joint using the flexor pollicis longus, indicating a positive Froment’s sign (positive on the left, negative on the right). (B) Crossed finger test. When the patient attempts to cross the middle finger over the index finger, weakness or inability indicates dysfunction of ulnar-innervated interosseous muscles (positive on the left, negative on the right).

The patient reported weakness in the left hand accompanied by tingling pain localized to the ulnar aspect, which developed after the spinal surgery. The pain intensity was rated as 3 on the numeric rating scale. On physical examination, manual muscle testing revealed decreased strength of the left abductor digiti minimi muscle, corresponding to Medical Research Council grade 3. Sensory examination revealed hypoesthesia involving the fifth digit and medial side of the fourth digit. Froment’s sign (Fig. 1A) and the crossed finger test (Fig. 1B) were positive on the left side but negative on the right side, indicating weakness of the left intrinsic hand muscles innervated by the ulnar nerve. Deep tendon reflexes of the biceps and triceps were normal and symmetric. Spurling’s test and Tinel’s sign at the wrist were negative, and no upper motor neuron signs, such as Hoffmann reflex, were observed. Provocative test results for thoracic outlet syndrome, including Roos and Adson’s tests, were also negative.

## Differential diagnosis

The following potential diagnoses were considered to explain the patient’s left-hand weakness and sensory disturbance.

### 1. Ulnar neuropathy at the elbow level

Ulnar neuropathy at the elbow level was considered the most likely diagnosis. Compression of the ulnar nerve at this level commonly produces numbness and tingling in the medial aspect of the hand, particularly affecting the fourth and fifth digits, along with weakness of the intrinsic hand muscles. In this patient, hypoesthesia of the fifth digit and medial side of the fourth digit, positive Froment’s sign and crossed finger test results, and weakness of the abductor digiti minimi were consistent with ulnar nerve dysfunction at the elbow level. Ulnar neuropathy commonly arises from lesions at the elbow level. In addition, the temporal association with recent prolonged spinal surgery indicates the possibility of intraoperative compression at the medial aspect of the elbow.

### 2. C8 radiculopathy

C8 radiculopathy was also considered because neuropathic pain from C8 nerve root involvement may radiate to the medial forearm and hand and is often associated with motor deficits in muscles innervated by the C8 nerve root. In our patient, this diagnosis was considered less likely given the absence of scapular and upper arm pain, negative Spurling’s test results, and preserved deep tendon reflexes. However, cervical radiculopathy remained in the differential diagnosis, as patients may present with pain localized to the medial aspect of the hand, even in the absence of other characteristic symptoms or positive physical examination findings.

### 3. Ulnar neuropathy at the wrist level (Guyon’s canal syndrome)

Ulnar neuropathy at the wrist level (Guyon’s canal syndrome) was included in the differential diagnosis as entrapment of the ulnar nerve within Guyon’s canal can cause sensory changes in the fourth and fifth digits, with weakness of the intrinsic hand muscles. However, the incidence of this syndrome is lower than that of ulnar neuropathy at the elbow level [1].

### 4. Thoracic outlet syndrome

Thoracic outlet syndrome was also considered. Neurogenic thoracic outlet syndrome can result from compression of the lower trunk of the brachial plexus, potentially presenting with pain and paresthesia along the medial aspect of the upper extremity, accompanied by intrinsic hand muscle weakness. Nevertheless, the absence of vascular symptoms and negative provocative tests, including the Roos and Adson tests, rendered this diagnosis unlikely.

### 5. Cervical myelopathy

Cervical myelopathy was considered, given the patient’s age and presence of hand weakness. However, this diagnosis was considered less likely given the absence of upper motor neuron signs such as hyperreflexia, pathological reflexes, or bilateral symptoms.

## Diagnosis

Electrodiagnostic testing demonstrated marked slowing of motor conduction velocity across the left ulnar nerve at the elbow, with measurements of 15 m/second 2 cm proximal to the medial epicondyle, 29 m/second at the epicondyle level, and 29 m/second 2 cm distal to the epicondyle. Compound muscle action potential amplitudes were reduced, measuring 2.2 mV and 1.6 mV at the wrist and elbow, respectively. A sensory nerve conduction study demonstrated a decreased sensory nerve action potential amplitude of 13 μV at the wrist. Needle electromyography showed abnormal spontaneous activity, characterized by fibrillation potentials and positive sharp waves, in the left abductor digiti minimi and first dorsal interosseous muscles. These findings indicate active denervation, reflecting demyelination and axonal involvement of the ulnar nerve at the elbow level. Cervical magnetic resonance imaging revealed no abnormalities.

Based on the clinical presentation and electrodiagnostic findings, the patient was diagnosed with left ulnar neuropathy at the elbow level. Given its temporal relationship with prolonged spinal surgery, intraoperative compression at the medial elbow was considered the most likely etiology.

## Treatment and prognosis

An ultrasound-guided perineural injection of triamcinolone (10 mg) was administered to the left ulnar nerve at the elbow. One week after the intervention, the patient reported improvement in the tingling pain, with the numeric rating scale score decreasing from 3 to 1. However, no improvement in motor weakness was observed.

## Discussion

Ulnar neuropathy at the elbow is one of the most common entrapment neuropathies of the upper extremities and is frequently associated with prolonged elbow flexion or external compression [2]. Peripheral nerve injuries may occur because of prolonged surgical positioning [3]. In our case, the onset of symptoms after spinal surgery suggested that sustained compression at the medial elbow during the operative period was the primary contributing factor.

The clinical features of the patient, characterized by intrinsic hand muscle weakness, sensory disturbance in the fifth digit and medial side of the fourth digit, and positive Froment’s sign and crossed finger test results, were consistent with ulnar neuropathy [1]. Electrodiagnostic studies were essential in localizing the lesion to the elbow, demonstrating focal conduction slowing across the medial epicondyle and evidence of denervation in the ulnar nerve-innervated muscles. These findings effectively distinguish ulnar neuropathy from C8 radiculopathy, cervical myelopathy, and more distal ulnar nerve lesions [4].

Froment’s sign and the crossed finger test are useful bedside maneuvers for detecting weakness in the ulnar-innervated intrinsic hand muscles [5]. Froment’s sign occurs when weakness of the ulnar-innervated adductor pollicis muscle causes the patient to compensate by flexing the thumb interphalangeal joint via the flexor pollicis longus. The crossed finger test evaluates the interosseous muscles that are responsible for finger abduction and scissoring functions mediated by the ulnar nerve. In our patient, both Froment’s sign and the crossed finger test results were positive on the left side, indicating intrinsic muscle weakness consistent with ulnar neuropathy at the elbow rather than a proximal cervical lesion.

In our patient, spinal surgery was performed in the prone position, and the close temporal association indicated that intraoperative compression at the medial elbow was the most likely cause of ulnar neuropathy. In prone spinal surgery, several factors can contribute to ulnar nerve injury, including direct pressure over the cubital tunnel, excessive elbow flexion, insufficient padding, malpositioned blood pressure cuffs, and accidental arm displacement [6]. Elbow flexion beyond 70° to 100° is associated with increased intraneural and extraneural compression, and flexion greater than 135° can cause significant nerve elongation and ischemic stress [6]. Therefore, preventive measures, such as avoiding excessive elbow flexion, maintaining the joint near neutral, and adequate protection of the medial epicondyle, are essential during prolonged prone procedures to minimize the risk of compression neuropathy in patients after surgery.

This case emphasizes the importance of considering perioperative peripheral nerve injury in patients who develop new neurological symptoms during postoperative rehabilitation. Early electrodiagnostic evaluation facilitates accurate diagnosis and enables appropriate management. Ultrasound-guided steroid injections may help alleviate neuropathic pain by reducing perineural inflammation. Careful attention to intraoperative positioning remains critical in preventing this potentially avoidable complication.

## Educational pearls

1. Ulnar neuropathy at the elbow is the most common site of ulnar nerve entrapment. It is often associated with prolonged elbow flexion or external compression and should be considered in patients presenting with hand weakness and sensory disturbance of the ulnar side of the hand postoperatively.

2. Electrodiagnostic studies are essential for localizing ulnar nerve lesions and differentiating elbow-level entrapment from distal lesions (Guyon’s canal syndrome) or proximal causes such as C8 radiculopathy, thoracic outlet syndrome, and cervical myelopathy.

3. Perioperative positioning is a modifiable risk factor. Careful attention during surgery is essential because prolonged elbow flexion or external compression can lead to intraoperative ulnar nerve injury.

## Question 1

A 70-year-old man developed weakness in his left hand and a tingling sensation along the ulnar side of the hand after prolonged spinal surgery. Physical examination showed decreased strength of the abductor digiti minimi, sensory loss over the fifth digit and medial fourth digit, and positive Froment’s sign and crossed finger test results on the left side. Spurling’s test and Tinel’s sign at the wrist were negative. Which of the following is the most likely diagnosis?

- A. C8 radiculopathy
- B. Ulnar neuropathy at the elbow
- C. Carpal tunnel syndrome
- D. Thoracic outlet syndrome
- E. Cervical myelopathy

B

## Question 2

During physical examination, the patient is asked to hold a piece of paper between the thumb and index finger while the examiner attempts to pull it away. To maintain grip, the patient flexes the interphalangeal joint of the thumb. This finding suggests which of the following?

- A. Median nerve injury resulting in the inability to oppose the thumb
- B. Radial nerve palsy affecting wrist and finger extensors
- C. Compensatory activation of the flexor pollicis longus owing to the weak adductor pollicis
- D. Normal response with intact pinch strength
- E. C8 radiculopathy causing medial forearm and finger weakness

C
